# Symmetry Breaking of Molecules Triggered by Chiral Inorganic Nanostructures Without Organic Components

**DOI:** 10.1002/advs.202504269

**Published:** 2025-06-20

**Authors:** Tao Yao, Xiao Chen, Yating Fang, Zhifeng Huang

**Affiliations:** ^1^ Department of Chemistry The Chinese University of Hong Kong Shatin, New Territories Hong Kong SAR 999077 P. R. China; ^2^ Shenzhen Research Institute The Chinese University of Hong Kong No.10, 2nd Yuexing Road, Nanshan Shenzhen Guangdong Province 518057 P. R. China

**Keywords:** chiral nano‐inorganics, circularly polarized light, electron spin, enantiospecific interactions, symmetry breaking

## Abstract

Chirality transfer has garnered significant attention due to its potential to revolutionize asymmetric synthesis of enantiopure compounds and to provide fundamental insights into molecular symmetry breaking, which is widely believed to underlie the pervasive phenomenon of homochirality in biological systems. While organic‐to‐organic and organic‐to‐inorganic chirality transfers have been extensively studied, the inorganic‐to‐organic counterpart remains challenging due to the dimensional mismatch between the nanoscale of inorganic materials and the molecular scale of organic compounds, the thermodynamic instability of chiral inorganic lattices, and the uncontrollable influence of defects on chirality transfer. This review summarizes the synthesis of organic‐free chiral nano‐inorganic materials and explores the concept of inorganic‐to‐organic chirality transfer via symmetry breaking induced by these materials through three proposed pathways: enantiospecific inorganic–organic interactions, chirality transfer from circularly polarized light to molecules, electron spin‐determined enantiopreferential reactions governed by chiral‐induced spin selectivity. Inorganic‐to‐organic chirality transfer via symmetry breaking offers a novel framework for investigating spin chemistry, advancing asymmetric synthesis, enabling enantioselective production of single‐enantiomer pharmaceuticals, and elucidating the origin of homochirality on prebiotic Earth.

## Graphical abstract

Chiral nano‐inorganics, free of organic chiral ligands, provide a novel platform to direct symmetry breaking in molecular systems, through inorganic‐to‐organic chirality transfer via enantiospecific inorganic–organic interactions, circularly polarized light, and electron spin‐driven enantiopreferential reactions governed by chiral‐induced spin selectivity. These chirality‐transfer mechanisms offer profound insights into asymmetric synthesis, spin chemistry, and the origin of biological homochirality.

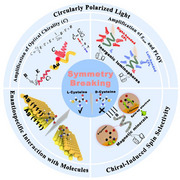

## 1 Introduction

Chirality, defined as an asymmetric configuration property whereby an object cannot be superimposed on its mirror image, is a ubiquitous phenomenon observed in cosmic systems, materials, structures, and biological systems.^[^ ^1^ ^]^ A pair of chiral objects are referred to as enantiomorphs, while enantiomer designates one member of a pair of chiral molecules.^[^ ^2^ ^]^ Biological systems exhibit unique characteristic known as homochirality, whereby biological building blocks (such as amino acids and sugars) exist almost exclusively in one of two chiral absolute configurations.^[^ ^3^ ^]^ Homochirality underlies a broad spectrum of asymmetric biochemical processes, including enzyme catalysis, receptor‐ligand binding, and the formation of complex molecular structures that dictate biological activities and functions.^[^ ^4^ ^]^ The pervasive presence of homochirality in biological systems has profound implications of molecular recognition and biochemical specificity, with far‐reaching relevance to public health, environmental pollution, and sustainability development.^[^ ^5^ ^]^

To fully understand and harness the homochirality‐governed asymmetric bio‐systems, it is of fundamental importance to control and manipulate the molecular handedness,^[^ ^6^ ^]^ namely, through enantioselective (or asymmetric) synthesis, which represents a central topic in modern chemistry and a key technique for producing enantiopure chiral pharmaceuticals.^[^ ^7^ ^]^ Conventional enantioselective methods primarily rely on chirality transfer, wherein chiral information is transmitted from one entity to another. In traditional asymmetric catalysis, this typically entails the transfer of chirality from a chiral organic molecule (e.g., a chiral ligand) to a prochiral or racemic substrate, thereby favoring the preferential formation of one enantiomer over the other. This process occurs via homogeneous or heterogeneous symmetry breaking (referred to a process whereby the symmetry of a system is lost or changed, resulting in the emergence of an enantiopreferential configuration) of molecules in asymmetric catalysis.^[^ ^8^ ^]^

The underlying mechanisms of symmetry breaking can be broadly interpreted from thermodynamic and kinetic perspectives. From the thermodynamical point of view, symmetry breaking can be described as a transition from a symmetric state to one of two chiral minima on the free energy landscape, driven by internal fluctuations or external chiral fields that break mirror symmetry. Kinetically, chiral preference can emerge through asymmetric reaction pathways, such as chiral autocatalysis or differential activation energies for competing enantiomeric routes. In solid‐state catalysis, surface‐induced chiral fields can bias molecular adsorption and transition states, leading to asymmetric product distributions even in the absence of chiral ligands. Together, these thermodynamic and kinetic models offer a conceptual foundation for understanding the enantiopreferential emergence of molecular handedness from symmetry.^[^ ^9^ ^]^

Traditionally, symmetry breaking is homogeneously catalyzed by metal cations bound to chiral ligands, through chirality transfer from chiral ligands to organic products.^[^ ^10^ ^]^ Such organic‐to‐organic chirality transfer can also occur in a heterogeneous manner, through symmetry breaking on solid‐state catalysts composed of achiral metals (e.g., as metal atoms, nanoclusters, and nanoparticles), which are grafted with chiral ligands.^[^ ^11^ ^]^ Since 2000, chiral metal nanoclusters were fabricated through chirality induction by chiral ligands, and the as‐generated chiral nano‐metals are inevitably terminated with chiral ligands.^[^ ^12^ ^]^ Such hybrid combination of chiral ligands and metals promisingly proffers a next generation of asymmetric catalysts,^[^ ^13^ ^]^ which are new venues to direct symmetry breaking of molecules. However, symmetry breaking rarely proceeds through chirality transfer from chiral nano‐metals themselves and instead occurs predominantly through the attached chiral ligands. It is mainly ascribed to the better dimensional compatibility between molecular substrates/intermediates and chiral ligands, relative to nanometal surfaces.

In the 2010s, chiral nano‐inorganics free of chiral organic components—namely, inorganic nanostructures exhibiting the chirality without the aid of organic molecules such as chiral ligands, templates, or additives—were successfully synthesized, as previous reviewed.^[^ ^14^ ^]^ Lack of chiral ligands in chiral nano‐inorganics enables the investigation of the inorganic‐to‐organic chirality transfer, forming the basis for recently developed strategies to manipulate inorganic‐induced symmetry breaking for enantioselective synthesis, even though the investigation is in its infancy.^[^ ^15^ ^]^ This review provides an updated overview of various methods for producing organic‐free chiral nano‐inorganics. Subsequently, three types of chiral stimulations to trigger symmetry breaking, associated with chiral nano‐inorganics, will be elucidated, including enantiospecific inorganic–organic interactions, circularly polarized light (CPL), electron spin‐determined enantiopreferential reactions governed by chiral‐induced spin selectivity (CISS). The underlying mechanisms by which these external chiral stimuli induce symmetry breaking will be analyzed, along with preliminary demonstrations. Finally, we offer perspectives to promote the development of symmetry breaking induced by organic‐free chiral nano‐inorganics, providing an essential insight into understanding the origin of homochirality and shedding light into devising additional methodologies for efficient manipulation of symmetry breaking—an essential step toward the production of enantiopure pharmaceutics.

## 2 Fabrication of Chiral Nano‐Inorganics Free of Chiral Ligands

The synthesis of organic‐free chiral nano‐inorganics—a prerequisite for investigating intrinsic chirality transfer from inorganics to organics—relies on two principal strategies: spontaneous symmetry breaking during crystallization and externally induced symmetry breaking.

**Figure 1:**
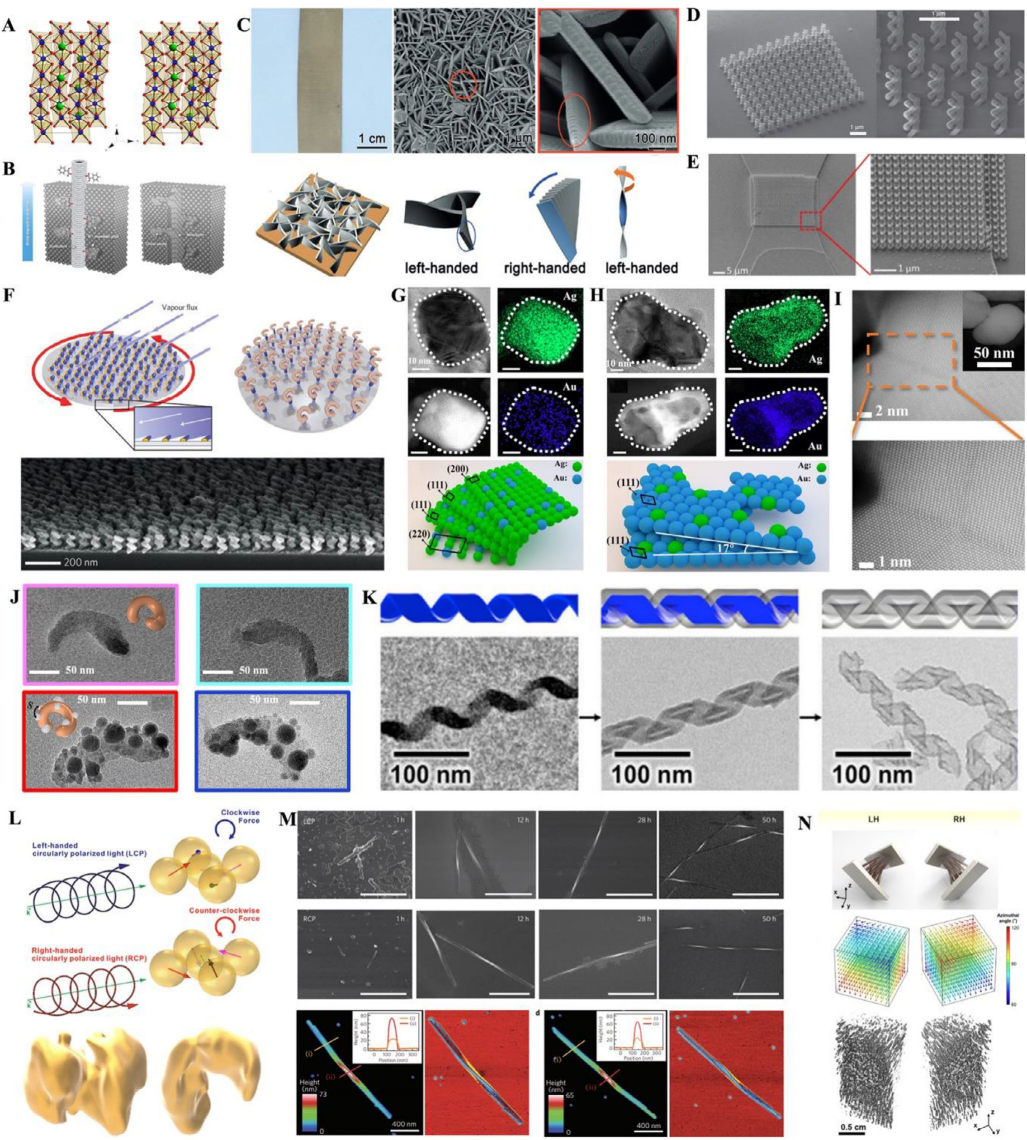
Fabrication of chiral nano‐inorganics without chiral ligands. A) Chiral crystal structures of CsCuCl_3_ in right‐handed (space group: P6_1_22, on the left side) and left‐handed (space group: P6_5_22, on the right side). B) Enantiomer‐imprinted mesoporous platinum (Pt) thin films, followed by removal of the imprinted enantiomers. C) Silver (Ag) films with hierarchical chirality. D) Triple‐helical nanowires generated by focused ion beam‐induced deposition, associated with tomographic rotatory growth. E) A Pt/carbon nanohelix array fabricated by focused electron beam‐induced deposition. F) Gold (Au) NHs deposited by glancing angle deposition on a periodic array of seeds. Binary Ag:Au chiral NPs G) without and H) with mesoporous structures, composed of chiral twisting of multiple achiral facets. I) Ag chiral NPs with chiral defects at the cores of chiral twisting twin boundaries. J) Helical assembly of gallium (Ga) NPs on silica NHs serving as a chiral template. K) Silica chiral nanoribbons grown on an organic chiral template (C_2_H_4_‐1,2‐((CH_3_)_2_N^+^C_16_H_33_)_2_ with a tartrate counterion), followed by removal of the chiral template. CPL‐induced formation of L) Au chiral NPs and M) CdTe nanospirals. N) Assembly of helical magnetoplasmonic nanochains induced by helical magnetic fields. A) Adapted with permission.^[^ ^17^ ^]^ Copyright 2024, IOP Publishing. B) Adapted with permission.^[^ ^20^ ^]^ Copyright 2016, Springer Nature. C) Adapted with permission.^[^ ^21^ ^]^ Copyright 2017, Wiley. D) Adapted with permission.^[^ ^22^ ^]^ Copyright 2015, Springer Nature. E) Adapted with permission.^[^ ^23^ ^]^ Copyright 2023, Wiley. F) Adapted with permission.^[^ ^24^ ^]^ Copyright 2013, Springer Nature. G, H) Adapted with permission.^[^ ^25^ ^]^ Copyright 2023, Royal Society of Chemistry. I) Adapted with permission.^[^ ^26^ ^]^ Copyright 2018, Wiley. J) Adapted with permission.^[^ ^27^ ^]^ Copyright 2022, Wiley. K) Adapted with permission.^[^ ^28^ ^]^ Copyright 2016, American Chemical Society. L) Adapted with permission.^[^ ^29^ ^]^ Copyright 2019, American Chemical Society. M) Adapted with permission.^[^ ^30^ ^]^ Copyright 2014, Springer Nature. N) Adapted with permission.^[^ ^31^ ^]^ Copyright 2020, American Chemical Society.

Among the 230 possible crystallographic space groups, only 22 are chiral—comprising 11 enantiomorphous pairs—making spontaneous symmetry breaking during crystallization inherently rare. For example, crystallization in an aqueous solution with stirring led to the formation of NaClO_3_ crystals in either levorotatory or dextrorotatory of P2_1_3 space group.^[^ ^16^ ^]^ Similarly, stirring‐driven solution crystallization of CsCuCl_3_ yields enantiopure single crystals in either the right‐handed P6_1_22 or left‐handed P6_5_22 (**Figure** **1**A).^[^ ^17^ ^]^ However, such stirring‐induced crystallization results in stochastic—rather than deterministic—symmetry breaking.

Chirality transfer from external chiral forces to inorganics has been widely employed to produce chiral nano‐inorganics. Organic chiral ligands were intuitively used to transfer molecular chirality to inorganic materials,^[^ ^18^ ^]^ after which their removal yields organic‐free chiral nano‐inorganics.^[^ ^19^ ^]^ For instance, enantiomers were imprinted in metallic thin films during the electrochemical deposition of metals in the presence of enantiomers, and subsequent removal of the imprinted enantiomers results in metallic cavities that retain the enantiomeric configuration (Figure 1B).^[^ ^20^ ^]^ Such organic‐to‐inorganic chirality transfer enables significant amplification of inorganic chirality, as demonstrated by the formation of silver (Ag) films exhibiting hierarchical chirality: primary spirally twisted nanoflakes; secondary helical stacking forming chiral nanoplates; and tertiary micrometer‐sized circinate structures composed of chirally arranged nanoplates (Figure 1C).^[^ ^21^ ^]^

**Figure 2:**
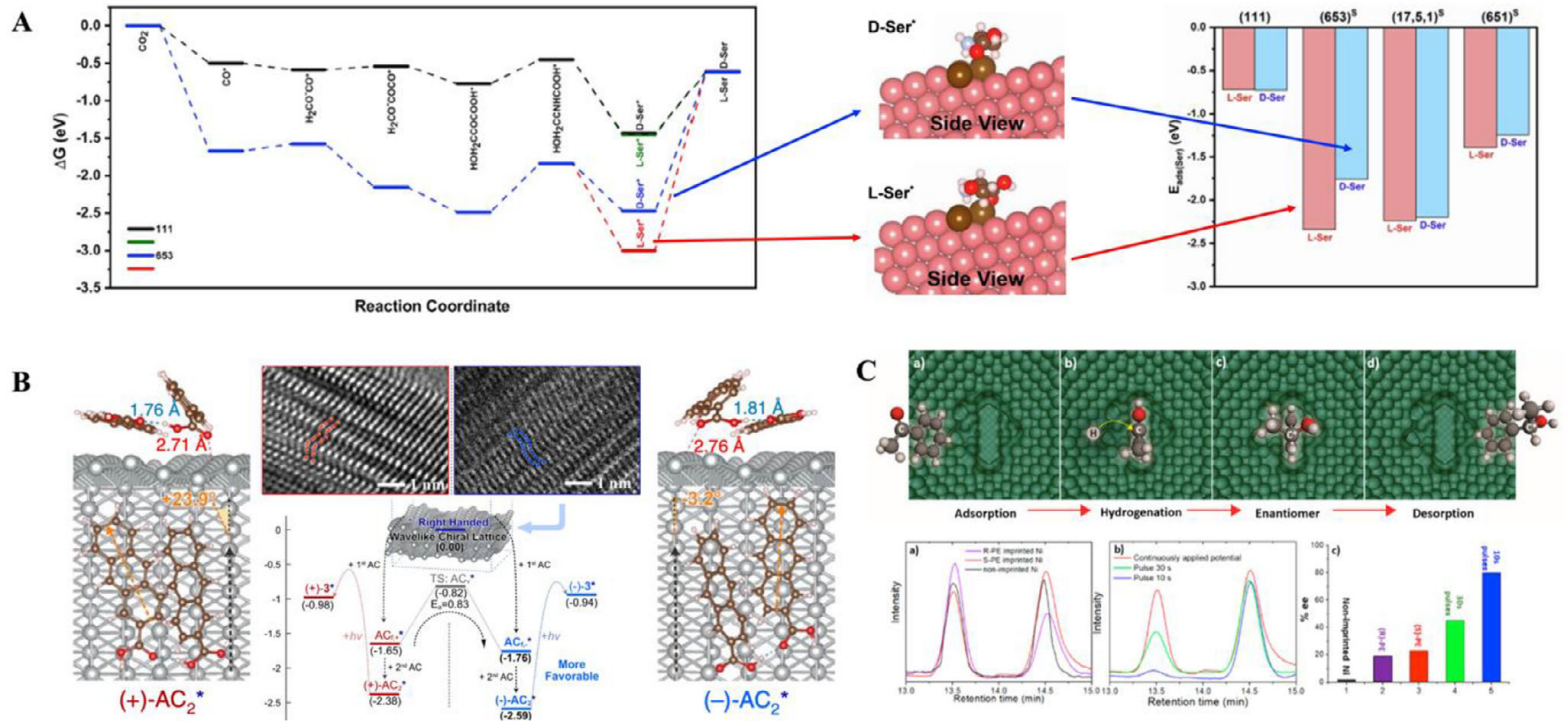
Symmetry breaking triggered by enantiospecific interactions between inorganic chiral lattices and molecules. A) Enantiopreferential production of L‐serine on the chiral kink sites Cu(653)*^S^* through the electrochemical catalysis of CO_2_ and NH_3_. B) Enantioselective photoinduced cyclodimerization of prochiral 2‐anthracenecarboxylic acid on the wavelike Ag chiral lattices. C) Enantioselective synthesis of phenylethanol (PE) via the electroreduction of acetophenone in the PE‐imprinted chiral Ni cavities. A) Adapted with permission.^[^ ^40^ ^]^ Copyright 2022, Elsevier. B) Adapted with permission.^[^ ^9^ ^]^ Copyright 2020, Springer Nature. C) Adapted with permission.^[^ ^41^ ^]^ Copyright 2019, American Chemical Society.

Beyond the chirality duplication and induction by chiral ligands, chirality transfer to inorganic materials has also been achieved using alternative chiral stimuli. During the deposition of inorganics on a supporting substrate powered by focused ion beams (Figure 1D),^[^ ^22^, ^32^ ^]^ focused electron beams (Figure 1E),^[^ ^23^ ^]^ and electron beam evaporation (Figure 1F),^[^ ^33^ ^]^ controlled rotation of the substrate or precursor sources in clockwise and counterclockwise enables the formation of inorganic nanohelices (NHs) composed of the nano‐scale helicity in right‐handed (RH) or left‐handed (LH). The formation of NHs originates from macroscopic shear forces induced by the mechanic rotation, accompanied by the translation of evaporated inorganics along the rotation axis (Figure 1F).^[^ ^24^ ^]^ Acceleration of substrate rotation can effectively impose the atomic‐scale handedness onto metals, which are composed of chiral twisting of multiple achiral facets (Figure 1G,H),^[^ ^25^ ^]^ chiral defects located at the cores of chiral twisting twin boundaries (Figure 1I),^[^ ^26^ ^]^ and wavelike chiral lattices (**Figure** **2**B).^[^ ^9^ ^]^ Galvanic replacement of metallic chiral lattices with heterogenous metal atoms results in the formation of alloy chiral nanoparticles (NPs)^[^ ^34^ ^]^ possessing mesoporous architectures (Figure 1H).^[^ ^25^, ^35^ ^]^ The handedness of NHs, functioning as a chiral template, was effectively transferred to inorganic NPs assembling on the chiral templates (Figure 1J,K).^[^ ^27^, ^28^ ^]^ Chirality transfer induced by CPL was applied to gold (Au) NPs to form chiral nanostructures (Figure 1L),^[^ ^29^, ^36^ ^]^ and to drive the self‐assembly of CdTe nanospirals, whose handedness was enantiopreferentially determined by the handedness of CPL (Figure 1M).^[^ ^30^ ^]^ Furthermore, magnetic NPs were aligned with chiral magnetic fields to assemble into chiral chains, as a result of magnetic dipole–magnetic field interactions (Figure 1N).^[^ ^31^ ^]^ Chirality transfer driven by the diverse external chiral stimuli effectively imprints chirality onto inorganic nanostructures across multiple dimensional scales—including atomic, molecular, nano, and microscale levels.

Three types of chiral forces, which are essentially associated with organic‐free chiral nano‐inorganics to trigger symmetry breaking, are discussed as follows.

## 3 Enantiospecific Interactions Between Inorganic Chiral Lattices and Molecules

When chiral or prochiral molecular substrates or intermediates approach the inorganic surfaces composed of atomic‐scale chiral lattices,^[^ ^37^ ^]^ molecule‐inorganic enantiospecific interactions result in enantiopreferential adsorption of molecules with an absolute configuration over its enantiomeric counterpart, i.e., symmetry breaking of adsorbents.^[^ ^38^ ^]^ The chiral/prochiral adsorbents with an absolute configuration have the adsorption energy lower than another. According to the Boltzmann distribution, at room temperature the low‐adsorption‐energy enantiomers will be more than their mirror images in a few orders of magnitude at the chiral inorganic surfaces, accounting for the enantiopreferential molecular adsorption. Additionally, inorganic chiral lattices can induce polarization or delocalization of electronic orbits and variation in electron spin states.^[^ ^39^ ^]^

For example, an electrodeposition of copper (Cu) in the presence of L‐histidine, followed by the removal of L‐histidine, led to the generation of chiral kink sites Cu(653)*^S^* at the Cu surfaces, where L‐serine was enantioselectively produced with an enantiomeric excess (e.e.) value larger than 90% through an electrochemical catalysis of CO_2_ and NH_3_ (**Figure** **2**A).^[^ ^40^ ^]^ As revealed by numerical simulations, the formation of chiral 3‐hydropyruvic acid from the intermediates H_2_CO‐CO* is the stereo‐determining step, whereby the enantiospecific stereo‐alignment of the hydroxymethyl and carboxyl groups with the chiral kink sites of Cu(653)*^S^* accounts for the enantiopreferential formation of L‐serine. Theoretical calculations further revealed that L‐serine exhibits a lower adsorption energy on the Cu(653)*^S^* surface compared to D‐serine, indicating that the asymmetric adsorption energies on chiral Cu surfaces play a crucial role in symmetry breaking.

Another example involves the handedness of wavelike Ag chiral lattices, which enantioselectively determines the absolute configuration of cyclodimers produced through the photoinduced cyclodimerization of prochiral 2‐anthracenecarboxylic acid (or AC).^[^ ^9^ ^]^ Numerical simulations show that AC molecules are stereoselectively adsorbed at the wavelike Ag chiral lattices to form enantiomorphous *anti*‐head‐to‐head dimers AC_2_*. The RH‐wavelike Ag chiral lattices enantiospecifically favor the formation of *Re*‐*Re* facial‐stacking (–)‐AC_2_*, which is more stable than the enantiomeric *Si*‐*Si* facial‐stacking (+)‐AC_2_*, as evidenced by its lower binding energy determined by the dimer configuration‐determined adsorption (Figure 2B). Consequently, the photoinduced cyclodimers (–)**‐3** are enantiopreferentially generated over their enantiomeric counterparts (+)**‐3** at the RH‐ Ag surfaces; vice versa, the LH‐wavelike Ag chiral lattices enable the enantioselective generation of (+)**‐3**. Not only Ag but also Cu chiral lattices result in the asymmetric photocyclodimerization of AC. This provides further evidence that asymmetric adsorption energies of enantiomers on chiral lattices are a key factor to drive symmetry breaking.

Furthermore, the enantiomer‐imprinted metal cavities (Figure 1B) enable one to enantioselectively tailor the absolute configuration of molecular products, through an electrochemical reduction of prochiral molecular precursors trapped in the chiral cavities. For instance, imprinting platinum (Pt) thin films with (*R*)‐mandelic acid (or MA) resulted in electrochemical reduction of phenylglyoxylic acid (PGA) within the (*R*)‐cavities and thus enantiopreferential generation of (*R*)‐MA over (*S*)‐MA with an e.e. value of up to 19%, owing to the sterically asymmetric hydroaddition of PGA determined by the (*R*)‐cavities where PGA molecules were accommodated.^[^ ^20^ ^]^ Conversely, (*S*)‐MA preferentially synthesized in the (*S*)‐cavities. Similarly, while imprinting nickel (Ni) thin films with (*S*)‐phenylethanol (or (*S*)‐PE) and (*R*)‐PE, the (*S*)‐ and (*R*)‐cavities led to an enantiopreferential generation of (*S*)‐PE and (*R*)‐PE, respectively, through the electrochemical reduction of acetophenone (Figure 2C).^[^ ^41^ ^]^ The e.e. value was amplified to as large as 80% using differential pulse voltammograms in the pulsed potential mode, and was further increased to 95% when the PE‐imprinted chiral electrodes were made of Pt‐iridium (Ir) alloys.^[^ ^42^ ^]^ Moreover, compared to unary (Pt) chiral electrodes, the binary ones significantly stabilized the electrochemically asymmetric production.

## 4 Circularly Polarized Light

**Figure 3:**
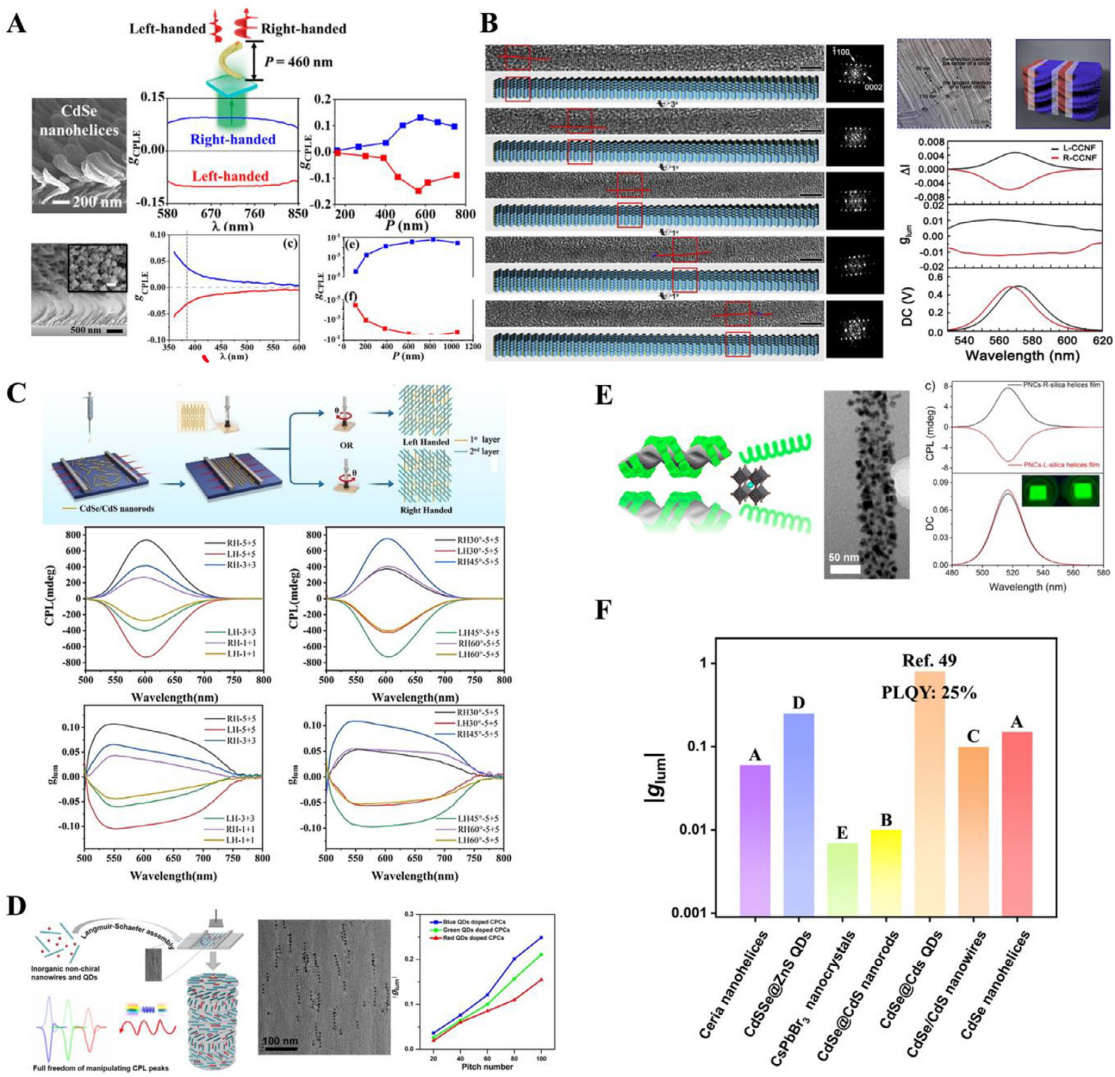
Circularly polarized light emitted from organic‐free chiral nano‐inorganics. A) CdSe and ceria NHs with a *g*_lum_ value significantly amplified with the engineering of helical pitch. B) CdSe@CdS nanorods composed of helical lattice distortion are helically assembled. Chiral photonic crystals: C) chiral twisting of multiple Langmuir thin films made of aligned CdSe/CdS nanowires; D) layer‐by‐layer Langmuir‐Schaefer chiral co‐assembly of NiMoO_4_ · *x*H_2_O nanowires and CdSSe@ZnS core@shell quantum dots. E) All‐inorganic perovskite CsPbBr_3_ nanocrystals are grafted on silica NHs. F) Summary of the |*g_lum_* | values reported for the organic‐free chiral nano‐inorganics, where the color of each column represents that of the generated CPL. A) Adapted with permission.^[^ ^44^ ^]^ Copyright 2023, American Chemical Society. B) Adapted with permission.^[^ ^46^ ^]^ Copyright 2021, Elsevier. C) Adapted with permission.^[^ ^47^ ^]^ Copyright 2021, Wiley. D) Adapted with permission.^[^ ^48^ ^]^ Copyright 2022, Wiley. E) Adapted with permission.^[^ ^51^ ^]^ Copyright 2020, American Chemical Society.

CPL serves as an external chiral bias to induce asymmetry in chemical systems by enantioselectively interacting with molecular electronic transitions. This interaction manifests through coupling chiral angular momentum of electromagnetic fields with electronic chiral orbitals of molecules, namely, differential absorption/excitation of RH‐CPL (or RCPL) and LH‐CPL (or LCPL) by molecular substrates/intermediates to realize chirality transfer from CPL to molecular products.^[^ ^43^ ^]^ Collectively, these effects disrupt the inherent energetic and kinetic equivalence of enantiomers in an achiral environment, to cause symmetry breaking (Figure 1L). Conventionally, CPL is generated by modulating non‐polarized incident through a half‐wave and quarter‐wave waveplate. Such complex optical system severely limits the on‐site applications of CPL. In this context, CPL emission from chiral nano‐inorganics represents a highly promising alternative. As recently reported, exposure of inorganic luminophore NHs to non‐polarized light results in the generation of CPL with a preferential polarization state dictated by their helical handedness: the LH and RH NHs preferentially emit RCPL and LCPL, respectively (**Figure** **3**A).^[^ ^44^ ^]^ It indicates the possibility for chiral nano‐inorganic luminophores to stimulate symmetry breaking through CPL, by providing a persistent and structurally encoded chiral field, offering a more accessible and robust platform for symmetry breaking in diverse chemical processes.

CPL emitted from chiral nano‐inorganics with and without chiral ligands has been comprehensively reviewed by Jiang and Kotov recently.^[^ ^45^ ^]^ It is worth to discussing the contribution of chiral ligands to the emission of CPL. Chiral ligands usually provide chiral perturbation in the local environment around the inorganic cores, and thus the emitted CPL are highly dependent on the conformational integrity and spatial orientation of the chiral organic moieties. In contrast, organic‐free chiral nano‐inorganics emit CPL owing to the asymmetric interactions of external excitation fields and their intrinsic structural chirality, and the emitted CPL is often more environmentally stable. In this review, we mainly focus on CPL generated from chiral nano‐inorganics without organic components, which can be categorized into two types: (1) inorganic luminophores with intrinsic chiral structures, and (2) achiral inorganic luminophores helically assembled on inorganic chiral templates. The former includes the sculpture of inorganic luminophores (such as CdSe and ceria, Figure 3A) in a helical shape using glancing angle deposition,^[^ ^44^ ^]^ and core@shell CdSe@CdS nanorods with helical lattice distortion due to the lattice mismatch (Figure 3B).^[^ ^46^ ^]^ The latter type includes chiral photonic crystals consisting of chiral twisting of multiple Langmuir thin films made of aligned CdSe/CdS nanorods (Figure 3C),^[^ ^47^ ^]^ layer‐by‐layer Langmuir‐Schaefer chiral co‐assembly of NiMoO_4_ · *x*H_2_O nanowires and CdSSe@ZnS quantum dots (Figure 3D),^[^ ^48^ ^]^ and all‐inorganic perovskite (CsPbBr_3_) nanocrystals grown on silica helical nanoribbons (Figure 3E).^[^ ^49^ ^]^ It should be noted that the inorganic luminophores are terminated with organic surfactants to prevent them from aggregation, and the organic surfactants make a negligible contribution to CPL. The CPL of chiral nano‐inorganics carries the cumulative effects of photons not only emitted from a chiral excited state of luminophores but also circularly scattered by the chiral nanostructures having the wavelength‐comparable helicity, known as circularly polarized luminescence and circularly polarized scattering, respectively.^[^ ^45^ ^]^

To facilitate the CPL‐to‐molecule chirality transfer, the quality of CPL should be enhanced with respect to three key factors. The first factor is circular polarization purity that can be quantitatively evaluated by a luminescence dissymmetry factor *g_lum_* =  2(*I_L_* − *I_R_*)/(*I_L_* + *I_R_*), where *I*_L_ and *I*_R_ represent the emission intensity of LCPL and RCPL, respectively. Exclusive emission of LCPL and RCPL corresponds to maximum *g_lum_* value of 2 and −2, respectively, while a racemic mixture of LCPL and RCPL results in a *g_lum_* value of 0. The larger the absolute *g_lum_* value (i.e., |*g_lum_* |), the purer the circular polarization of CPL. Chiral luminescent molecules generally have a |*g_lum_* | value of 10^−5^–10^−3^,^[^ ^50^ ^]^ and such poor circular polarization purity is substantially ascribed to the sub‐wavelength molecular sizes not comparable to the excitation wavelength. In contrast, chiral nano‐inorganics make the dimensional mismatch significantly ameliorated and result in the |*g_lum_* | values of 10^−3^–10^0^,^[^ ^45^ ^]^ which are summarized in Figure 3F. For those in a helical shape, the |*g_lum_* | values can be tuned with helical pitches (*P*). When perovskite CsPbBr_3_ nanocrystals were grafted on non‐luminous silica NHs with a *P* of ≈60 nm (which was significantly shorter than the excitation wavelength of 365 nm), the |*g_lum_* | value was 6 × 10^−3^ (Figure 3E).^[^ ^51^ ^]^ The hierarchically helical assembly of CdSe@CdS nanorods intrinsically composed of helical lattice distortion was formed with an unidentified, uncontrollable *P*, leading to the |*g_lum_* | value of as large as ≈0.01 at the excitation wavelength of 400 nm (Figure 3B).^[^ ^46^ ^]^ Excited at the wavelength at 320 nm, ceria NHs with a *P* of ≈830 nm has a |*g_lum_* | value of 0.06, which is amplified in 10^3^ folds compared to those with a *P* of ≈110 nm (Figure 3A).^[^ ^44^ ^]^ Under the 532‐nm excitation, CdSe NHs have a |*g_lum_* | value of 0.15 at a *P* of ≈570 nm, showing a 40‐fold amplification compared with those at a *P* of ≈160 nm. The chiral twisting of multiple achiral thin films (composed of luminescent inorganic nanostructures in an ordered assembly) enables the formation of chiral photonic crystals, which transmit CPL with a circular polarization determined by the handedness of chiral photonic crystals and reflect CPL with another circular polarization. The |*g_lum_* | values are essentially controlled by the overlap of band gaps between chiral photonic crystals and inorganic luminophores. The photonic band gaps were tuned by manipulating the numbers of Langmuir thin films and the interlayer twisting angles,^[^ ^47^ ^]^ the twisting *P*,^[^ ^48^ ^]^ and the assembled multilayer structures, to achieve a |*g_lum_* | value of as high as 0.8.^[^ ^52^ ^]^ The color of CPL is mainly determined by inorganic luminophores, and the columns shown in Figure 3F are represented in a color of the generated CPL.

The second factor is photoluminescence quantum yield (PLQY), defined as the ratio of emitted photons to absorbed photons. Large PLQY is favored to generate CPL with high intensity. PLQY is primarily governed by the absorption processes and radiative recombination rates of excitons, which are strongly influenced by the material composition and surface engineering of inorganic luminophores. To the best of our knowledge, only one study has reported PLQY measurements for organic‐free chiral nano‐inorganics: chiral photonic crystals composed of multilayers of the mixture of CdSe@CdS quantum nanorods (having an average diameter of 5 nm) and NiMoO_4_∙H_2_O nanowires, which exhibited a PLQY of 25%.^[^ ^52^ ^]^ The rationales for employing the core@shell quantum nanorods to obtain high PLQY are as follows: (1) The shell nanostructures effectively passivate defects at the core surfaces to prohibit the photoexcited excitons from nonradiative recombination; (2) strong Auger coupling at the core@shell interface facilitates fast nonradiative decay pathways that contribute to enhancing PLQY;^[^ ^53^ ^]^ (3) the quantum‐size nanorods tend to induce strong confinement of the photoexcited excitons and thus prevent the nonradiative recombination.^[^ ^54^ ^]^

Figure of merit (FOM) has been proposed to comprehensively evaluate the emission of CPL, given by *FOM*  =  |*g_lum_* |  ×  *PLQY*.^[^ ^55^ ^]^ Theoretically, pure circular polarization (with the maximum |*g_lum_* | value of 2) and sufficient photon conversion (with the maximum PLQY value of 1) will result in the maximum FOM value of 2. The chiral photonic crystals have the |*g_lum_* | value of 0.8 and PLQY of 25%,^[^ ^52^ ^]^ so that FOM = 0.2. It should be noted that the determinants of the |*g_lum_* | values usually decouple from those of the PLQY values, and thus these two CPL‐associated parameters could be separately tuned to facilitate the optimization of the FOM values.

**Figure 4:**
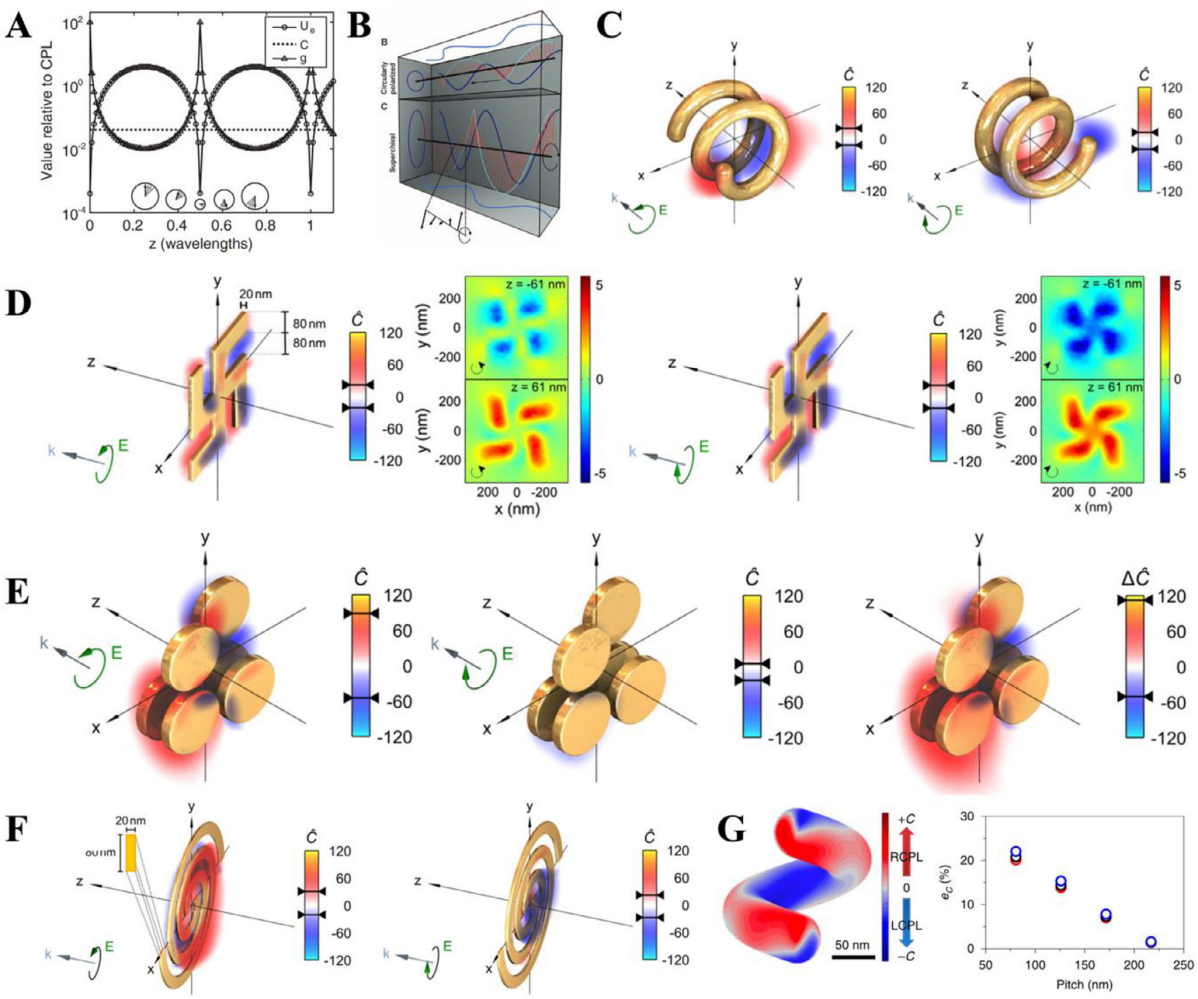
Circularly polarized light with amplified optical chirality *C*: **superchiral near fields**. A) Enhanced chiral asymmetry in superchiral light. The intensity (circles) is modulated in a standing wave, while the *C* (dashed line) is not. Amplification of *C* for B) LCPL standing wave, C) a LH helix exposed to LCPL and a RH helix exposed to RCPL at a wavelength of 2.03 µ*m*, D) a planar gammadion structure illuminated with LCPL and RCPL at a wavelength of 2.01 µ*m*, E) a chiral oligomer incident with LCPL and RCPL at a wavelength of 900 nm, F) a two‐armed gold nanospiral illuminated with LCPL and RCPL at a wavelength of 1.84 µ*m*, and G) a LH nanohelix incident with non‐polarized light (at a wavelength of 365 nm) as a function of helical pitch. A) Adapted with permission.^[^ ^57^ ^]^ Copyright 2010, American Physical Society. B) Adapted with permission.^[^ ^59^ ^]^ Copyright 2011, American Association for the Advancement of Science. C‐F) Adapted with permission.^[^ ^58^ ^]^ Copyright 2012, American Physical Society. G) Adapted with permission.^[^ ^9^ ^]^ Copyright 2020, Springer Nature.

The third factor is the optical chirality *C* of CPL, associated with the intrinsic mismatch between the wavelength of CPL (typically in the UV–vis region) and the sub‐wavelength dimensions of molecular substrates, which severely weakens the CPL‐molecule coupling. Such dimensional mismatch can be relieved to some extent by amplifying the non‐vector *C* of CPL, as defined by $C\equiv \frac{\in_{0}}{2}E\cdot \nabla \times E+\frac{1}{2\mu_{0}}B\cdot \nabla \times B$, where ∈ _0_ and µ_0_ are the permittivity and permeability of free space, respectively.^[^ ^56^ ^]^ E and B are the time‐dependent electric and magnetic fields, respectively. The quantity *C* describes the degree of chiral asymmetry in the rate of excitation of a chiral object (such as enantiomers) under an illumination with LCPL and RCPL. In other words, larger *C* value favors greater differential absorption of LCPL and RCPL by a given enantiomer.^[^ ^57^ ^]^ When irradiated with CPL under on‐resonant conditions, a chiral plasmonic (or chiroplasmonic) nanostructure (e.g., made of metals) will generate superchiral near fields via light absorption and then scattering in its vicinity, exhibiting an amplification of *C* that can be quantitatively evaluated by an enhancement factor $\hat{C}=C/C_{i}\;$, where *C_i_* represents the optical chirality of the incident CPL.^[^ ^58^ ^]^ $|\hat{C}|>1$ illustrates that compared with the incident CPL, the superchiral near field has an amplified *C* to partially reduce its wavelength and relieve the substantial mismatch between the CPL wavelength and molecular size (**Figure** **4**A,B).^[^ ^57^, ^59^ ^]^ Furthermore, $\hat{C}>0$ illuminates that the superchiral near field has a circular polarization state (LCPL or RCPL) the same as the incident CPL, and being opposite to the incident CPL leads to $\hat{C}<0$ (Figure 4C). The superchiral near fields have been theoretically predicted to be formed in the vicinity of chiroplasmonic nano‐metals with diverse chiral structures (Figure 4C–F).^[^ ^58^ ^]^ For example, it is assumed that the chiral intermediates generated in the electrochemical formation of amino acids (Figure 2A) have a preferential absorption of LCPL over RCPL at a given illumination wavelength. If the chiral intermediates are adsorbed on a chiral nano‐metal and the incident LCPL resonantly generates the superchiral near‐fields with $\hat{C}>1$ at the given illumination wavelength, the amplified superchiral LCPL will enantiopreferentially excite the adsorbed chiral intermediates and accelerate the formation of the homochiral amino acids through charge transfer mediated with chiroplasmonic hot electrons. The amplification of *C*, characterized with $\hat{C}$, will be facilely tuned with the helicity of chiroplasmonic nano‐metals, paving the way to performing photoinduced asymmetric synthesis and enantioselective deracemization.

CPL, traditionally generated by passing a non‐polarized light through a half‐waveplate and a quarter‐waveplate, has been applied to perform asymmetric synthesis via symmetry breaking.^[^ ^60^ ^]^ To the best of our knowledge, however, the amplification in the FOM and *C* values of CPL induced by chiral nano‐inorganics has yet been experimentally applied to perform photoinduced asymmetric synthesis or enantioselective deracemization. Recently, Wei et al. found that under non‐polarized illumination at the wavelength of 365 nm, the photoinduced cyclodimerization of AC dimers adsorbed on Ag NHs generally led to an e.e. value slightly larger than those on Cu NHs, in a *P* range of 8–220 nm.^[^ ^9^ ^]^ On one hand, the 365‐nm illumination will resonantly trigger chiral nanoplasmonics in the vicinity of the Ag NHs^[^ ^61^ ^]^ and thus produce the superchiral near‐fields. In contrast, chiral nanoplasmonics of the Cu NHs occur at a wavelength of ≈600 nm,^[^ ^34^ ^]^ so that the superchiral near‐fields could not be resonantly generated by the 365‐nm illumination. On the other hand, as revealed by the numerical simulations, the non‐polarized illumination causes a mixture of LCPL and RCPL superchiral near fields located along the helical profile (Figure 4G). Although the on‐resonant superchiral near fields in the vicinity of Ag NHs will facilitate the enantioselective cyclodimerization of AC dimers, the mixture of LCPL and RCPL superchiral near fields will severely deteriorate the photoinduced enantiopreference. Therefore, under the non‐polarized 365‐nm illumination the Ag NHs with the on‐resonant superchiral near fields cause the photoinduced enantiopreference slightly better than the Cu NHs without the superchiral near fields. To fully exploit resonant superchiral near fields to enhance the photoinduced enantioselectivity, an CPL illumination should be resonantly applied to the chiroplasmonic NHs.

## 5 Spin Polarization of Electrons

Symmetry breaking triggered by electron spin can be ascribed to the CISS effect derived from a coupling between the linear momentum of an electron moving in a chiral electric potential and its spin.^[^ ^62^ ^]^ As a result, a moving electron will be spin‐polarized either parallel or antiparallel to its velocity, which is determined by the handedness of chiral medium.^[^ ^2^ ^]^ Spin‐dependent electron transport offers transformative potential for spin‐selective redox reactions and spin‐based chemical selectivity, moving beyond traditional reaction controls and promising new spin‐driven reaction pathways.

**Figure 5:**
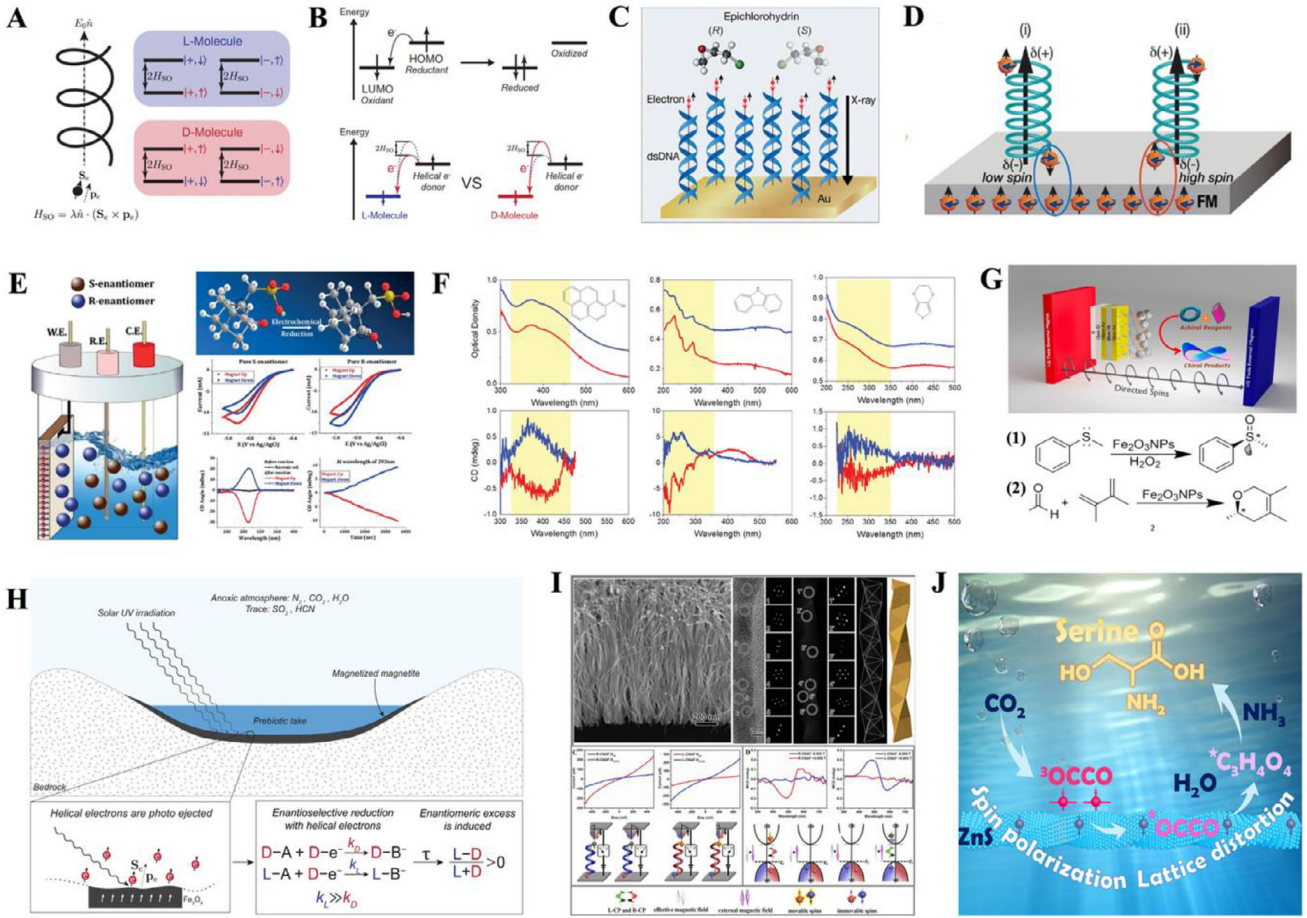
Symmetry breaking induced by spin polarization of electrons. A) Chirality induced spin selectivity (CISS) can be qualitatively ascribed to spin‐orbit coupling, with the spin‐orbit Hamiltonian *H_SO_*. B) The CISS‐induced spin‐selective reduction of molecules with a faster reduction rate by **exp**(2*H_SO_*/*k_B_T*). C) CISS‐determined deracemization of epichlorohydrin using spin‐polarized secondary electrons generated by X‐ray irradiation of an Au achiral thin film, where the self‐assembled homochiral monolayer of dsDNA on the Au film functions as a spin‐filter. D) Charge and spin polarization of a pair of enantiomers immobilized on a magnetized ferromagnetic surface. E) Enantioselective electro‐reduction of camphorsulfonic acid on the magnetized Ni electrodes. F) Asymmetric electro‐polymerization of achiral monomers on the magnetized ferromagnetic electrodes. G) Enantioselective reactions of achiral reactants performed on the magnetic ferromagnetic electrodes: oxidation of methyl phenyl sulfide to enantioselectively produce chiral methyl phenyl sulfoxide, and Diels−Alder cycloaddition of 2,3‐dimethylbutadiene with acetaldehyde. H) Spin‐selective photoinduced reduction at the surfaces of magnetized magnetite (Fe_3_O_4_) deposits in an evaporative lake. I) Chiral mesostructured Au with spin chiral anisotropy, in terms of electron transport and electron transition. J) CISS‐induced enantioselective synthesis of serine through photocatalysis of CO_2_ and NH_3_ at chiral mesostructured ZnS. A, B and H) Adapted with permission.^[^ ^63^ ^]^ Copyright 2022, National Academy of Sciences (USA). C) Adapted with permission.^[^ ^64^ ^]^ Copyright 2015, Wiley. D) Adapted with permission.^[^ ^65^ ^]^ Copyright 2018, American Association for the Advancement of Science. E) Adapted with permission.^[^ ^66^ ^]^ Copyright 2020, Wiley. F) Adapted with permission.^[^ ^67^ ^]^ Copyright 2020, Royal Society of Chemistry. G) Adapted with permission.^[^ ^68^ ^]^ Copyright 2021, American Chemical Society. I) Adapted with permission.^[^ ^75^ ^]^ Copyright 2023, American Chemical Society. J) Adapted with permission.^[^ ^76^ ^]^ Copyright 2025, Cell Press.

The CISS effect, in terms of electron transport in a chiral electric potential, can be qualitatively ascribed to the spin‐orbit coupling of the linear momentum ($p_{e}=m_{e}\;\upsilon$) of a moving electron (having the mass *m_e_* and velocity υ) with its spin angular momentum (*S_e_*), in a chiral electrostatic potential *E_chiral_* (=$E_{0}\;\hat{n}$, where $\hat{n}$ is pointing up and down in D‐ and L‐molecular frame, respectively, **Figure** **5**A).^[^ ^63^ ^]^ The moving electron in *E_chiral_* experiences an effective magnetic field $B=-\frac{1}{c^{2}}\upsilon \times E_{chiral}$, where *c* is the speed of light. The transporting electron with *S_e_* has the spin magnetic moment µ_*e*_ = −*g*µ_*B*_ *S_e_*/ℏ  (where *g*(≅2) is the spin *g* factor, µ_*B*_ is the Bohr magneton, and ℏ is the reduced Planck constant). The interaction of *B* with µ_*e*_ leads to splitting the electron energy represented by the spin‐orbit Hamiltonian $H_{SO}=-\mu_{e}\cdot B=g\mu_{B}E_{0}/\left(\hbar m_{e}c^{2}\right)\;\hat{n}\cdot \left(S_{e}\times p_{e}\right)$. In the molecular frame, an electron can move up (+) or down (‐), and spin up (↑) or down (↓), resulting in four energy states of | +, ↑〉, | −, ↓〉, | +, ↓〉, and | −, ↑〉. According to *H_SO_*, in the L‐molecule the moving‐up electron prefers to spin up (i.e., | +, ↓〉 −  | +, ↑〉 =  2*H_SO_*), and the moving‐down electron is favorable for spin down (i.e., | −, ↑〉 − | −, ↓〉 =  2*H_SO_*). In contrast, the moving‐up and moving‐down electrons prefer to spin down and spin up in the D‐molecule, respectively (Figure 5A). As a result, a L‐molecule will be preferentially reduced (with an electron moving up into the L‐molecule) by a spin‐up electron (through an intermediate state of | +, ↑〉) over by a spin‐down one (| +, ↓〉), with a faster reduction rate by exp(2*H_SO_*/*k_B_T*), where *k_B_* is the Boltzmann's constant and *T* is an absolute temperature (Figure 5B). In contrast, a spin‐down electron will reduce a D‐molecule faster than a spin‐up one. This is so‐called spin‐selective chemistry. It should be emphasized that the CISS effect will effectively prohibit the backscattering of spin‐polarized electrons transporting in chiral molecules, indicating that spin‐polarized electron transport in chiral molecules is more efficient than in achiral ones. However, the spin‐orbit coupling proposed in Figure 5A,B is too simple without considering the multi‐electron and electron‐phonon interactions, and thus the origin of the CISS effect is under debate.

The CISS‐induced spin‐selective reactions have been mainly performed on achiral inorganic electrodes, with the earliest example being the CISS‐mediated deracemization. In one such system, an Au thin film self‐assembled with double‐stranded DNA (dsDNA) was exposed to an X‐ray irradiation, the photoinduced secondary electrons transported from Au through the homochiral dsDNA with a preferential spin‐polarized state, whereby the homochiral dsDNA functioned as a spin filter owing to the CISS effect (Figure 5C).^[^ ^64^ ^]^ Then, also due to the CISS effect, the spin‐polarized electrons tended to enantioselectively reduce *S*‐epichlorohydrin over *R*‐epichlorohydrin at the dsDNA‐epichlorohydrin interfaces, leading to the deracemization of epichlorohydrin.

When an enantiomer adsorbs on a ferromagnetic inorganic surface, it becomes both charge‐ and spin‐polarized, with the spin polarization determined by the enantiomer's handedness and the direction of the substrate's magnetization. When the ferromagnetic substrate is magnetized up or down, one enantiomer is enantiopreferentially grafted on the magnetized ferromagnetic surface over its mirror image due to spin alignment constraints governed by the Pauli exclusion principle (Figure 5D).^[^ ^65^ ^]^ Such enantioselective adsorption of camphorsulfonic acid (CSA) results in the deracemization of CSA through electrochemical reduction of the enantiopreferentially adsorbed enantiomers on the magnetized ferromagnetic electrodes in a racemic solution of CSA (Figure 5E).^[^ ^66^ ^]^ Another example involves the CISS‐determined asymmetric synthesis, occurring through a reaction pathway that includes chiral intermediates. On a magnetized Ni/Au ferromagnetic electrode, an electropolymerization of achiral monomers leads to the formation of chiral polymers whose absolute configuration is controlled by a magnetic field applied up or down along the normal direct of the ferromagnetic electrode (Figure 5F).^[^ ^67^ ^]^ During the electropolymerization of achiral monomers, racemic chiral intermediates are generated. One of the pair of enantiomeric intermediates enantioselectively adsorbs on the magnetized electrode, where spin‐polarized electrons—dictated by the electrode's magnetization—are selectively injected into the adsorbed intermediate. This spin‐dependent interaction leads to differential reduction, enabling asymmetric synthesis of chiral polymers. Analogous method has been applied to the oxidation of methyl phenyl sulfide for the enantioselective formation of chiral methyl phenyl sulfoxide, and to Diels‐Alder cycloaddition of 2,3‐dimethylbutadiene with acetaldehyde to enantiopreferentially form 2,3,5‐trimethyl‐2,6‐dihydro‐2H‐pyran (Figure 5G).^[^ ^68^ ^]^ Spin polarization can be manipulated not only by external magnetic fields, but by the facets of magnetite minerals: spin‐down from the (111) facet and spin‐up from the (001) facet of magnetites (Figure 5H).^[^ ^63^ ^]^

To the best of our knowledge, limited efforts have been made to manipulate spin polarization using organic‐free chiral nano‐inorganics for triggering symmetry breaking. Recently, Prof. Che's group performed the chiral ligand‐induced hydrothermal syntheses (followed by the removal of chiral ligands) to fabricate chiral mesostructured inorganic materials (CMIMs), made of paramagnetic (Au^[^ ^69^ ^]^ and Au:Ag alloys^[^ ^70^ ^]^), antiferromagnetic (NiO^[^ ^71^ ^]^ and α‐Fe_2_O_3_^[^ ^72^ ^]^), ferrimagnetic (Fe_3_O_4_^[^ ^73^ ^]^ and γ‐Fe_2_O_3_^[^ ^72^ ^]^), and diamagnetic (BiOBr^[^ ^74^ ^]^) constituents. Paramagnetic CMIMs with high electrical conductivity exhibit the CISS‐determined electron transport, whereas antiferromagnetic and ferrimagnetic CMIMs show distinct spin chiral anisotropy (SChA) stimulated by external magnetic fields, making the CMIMs promisingly function as spin polarizers to induce symmetry breaking (Figure 5I).^[^ ^75^ ^]^ Currently, the CISS effect of the ZnS CMIMs was applied to perform the photocatalysis of CO₂ and NH₃, resulting in an enantioselective synthesis of serine with an e.e. value larger than 96% (Figure 5J).^[^ ^76^ ^]^ It was speculated that the excellent asymmetric photocatalysis could be attributed to the chiral induced spin polarization to facilitate the separation of photogenerated carriers and the production of stable ^3^OCCO intermediates. As proposed by Zhang et al., chiral nanostructured Ag films facilitate the spin‐selective charge transfer to stabilize the metastable triplet‐state intermediates ^3^OCCO.^[^ ^77^ ^]^ The metastable intermediates exhibit prolonged lifetime under asymmetric spin environments, which is critical for the enantiopreferential formation of C–N bonds.^[^ ^78^ ^]^ The spin‐polarized electrons generated by the CISS effect not only suppress the dissociation of ^3^OCCO but also steer its stereochemical alignment with NH₃, thereby amplifying the enantioselectivity. It should be noted that the post‐fabrication removal of chiral ligands could not guarantee the complete elimination of chiral ligands on the CMIMs, and traces of chiral residues will significantly mask the contribution of symmetry breaking from the CMIMs, because the enantiospecific interactions of chiral ligands and molecular substrates can independently induce symmetry breaking.

Overall, focusing on the organic‐free chiral nano‐inorganics not only expands the material scope for spin‐polarized technologies but also deepens our fundamental understanding of how structure, symmetry, and spin interplay in systems devoid of organic components. This inorganic‐centered perspective reinforces the viability of developing stable, tunable, and magnet‐free spintronic platforms driven by the CISS effect.^[^ ^79^ ^]^

## 6 Conclusion and Perspectives

Organic‐to‐organic and organic‐to‐inorganic chirality transfer, mediated with symmetry breaking, have been widely studied. Recently, it is significantly catching academic attention to investigate and realize inorganic‐to‐organic chirality transfer, with the aim of developing novel strategies for controlling molecular absolute configurations—one of the central challenges in modern chemistry. Achieving this objective, it is prerequisite to fabricate chiral nano‐inorganics free of organic chiral ligands. Currently, the prerequisite has been realized using diverse fabrication approaches, including the duplication of molecular chirality followed by the removal of imprinted chiral molecules, chirality transfer from organic chiral ligands followed by the removal of chiral ligands, symmetry breaking induced by clockwise/counterclockwise rotation or stirring, galvanic replacement of metal chiral lattices with heterogenous metal atoms, chiral assembly in inorganic chiral templates, CPL‐induced chirality transfer, and chiral assembly of magnetic NPs induced by chiral magnetic fields.

Symmetry breaking induced by organic‐free chiral nano‐inorganics plays a pivotal role in the inorganic‐to‐organic chirality transfer. Several mechanisms to achieve symmetry breaking have been discussed, including enantiospecific interactions between the atomic‐scale chiral inorganic topographies and molecular substrates/intermediates, the CPL‐to‐molecule chirality transfer, and the CISS effect. These symmetry‐breaking stimuli, induced by chiral nano‐inorganics, can give rise to either deracemization of a racemic mixture or enantiopreferential synthesis of enantiomers and polymers with desirable absolute configuration. However, the investigation in the generation of enantiopure functional molecular systems, through symmetry breaking driven by organic‐free chiral nano‐inorganics, is in its infancy, and the successful examples are limited (**Table** **1**). To further develop the limited studies and predict more efficient chirality transfer pathways, an integration of theoretical and computational approaches will be highly beneficial. For instance, density functional theory calculations can offer an atomic‐level insight into electronic structures and chiral interactions, molecular dynamics simulations can reveal the time‐evolving nature of inorganic–organic interfaces, and machine learning algorithms can be developed to predict optimal structural parameters of chiral nano‐inorganics to enhance the enantioselectivity through symmetry breaking. Incorporating these strategies will accelerate the rational design of next‐generation chiral inorganic systems with tailored symmetry‐breaking capabilities. To develop the limited studies, some perspectives are discussed as follows.

**Table 1:** Comparison of the e.e. values obtained through symmetry breaking induced by different chiral stimuli associated with the organic‐free chiral nano‐inorganics.

Chiral nano‐inorganics	Chiral stimuli to trigger symmetry breaking	|e.e.| (%)	Ref.
Ag nanohelices	inorganic–organic enantiospecific interactions	5.4	[9]
Cu nanohelices		3	
Chiral Cu surface		>90	[40]
Chiral imprinted mesoporous Ni		>80	[41]
Ferromagnetic electrode	Electron spin	9–44	[66]
Magnetized film		10–20	[67]
Hematite		1–8.5	[68]
Chiral mesostructured ZnS		96	[76]

### 6.1 Enantiospecific Inorganic–Organic Interactions

Inorganic–organic enantioselective interactions fundamentally require that inorganic surfaces comprise the atomic‐scale chiral lattices. These atomic‐scale chiral surfaces, primarily composed of metals and alloys, often serve as asymmetric catalysts capable of binding a wide range of molecular substrates or intermediates (Figure 2). However, it is well known that metals thermodynamically consist of achiral lattices; hence, chiral lattices of metals tend to be unstable under thermal, irradiative and electrochemical conditions. Upon exposure to high external energy, nano‐metals with the atomic‐scale chiral lattices inevitably undergo the chiral‐to‐achiral transformation, resulting in the loss of external chiral stimulation of symmetry breaking. For example, Ag chiral NPs with the wavelike chiral lattices completely degrade to achiral Ag NPs mainly composed of the (111) facets at 200 °C.^[^ ^80^ ^]^ Alloying Ag chiral NPs with aluminum (Al) significantly enhances the thermal stability of chiral lattices, whereby the complete chiral‐to‐achiral transformation is postponed to occur at 700 °C. Furthermore, alloying chiral nano‐metals with multiple elements can significantly increase the volume fraction of the atomic‐scale chiral lattices in the nano‐metals,^[^ ^81^ ^]^thereby enhancing the catalytical enantioselectivity. It illuminates that chiral nano‐alloys can potentially trigger symmetry breaking in a more enantiopreferential way than chiral unary nano‐metals.

### 6.2 CPL

CPL has been proposed to trigger symmetry breaking through chirality transfer from circularly polarized electromagnetic fields to chiral molecular orbitals. Although naturally sun light is non‐polarized, solar illumination of chiral nano‐inorganics enables the generation of CPL with a color essentially determined by the band gap of inorganic luminophores and circular polarization controlled by inorganic helicity. To enhance the CPL‐to‐molecule chirality transfer, it is vital to amplify the FOM values of CPL, i.e., simultaneous amplification of the |*g_lum_* | and PLQY values. However, the non‐polarized solar irradiation typically fails to generate superchiral near fields with strong optical chirality *C*, to prohibit one from obtaining high photoinduced enantioselectivity.

To simultaneously increase the values of |*g_lum_* |, PLQY and *C*, it is proposed to fabricate multiple‐shell chiral nano‐inorganics. For instance, inorganic NHs, serving as a chiral template, can be conformally coated with inorganic luminophores and then with plasmonic (such as Au and Ag) NPs, leading to the formation of the NHs@luminophore@NPs architectures. On one hand, the NHs@luminophore facilitates the emission of CPL with large |*g_lum_* | and PLQY values under irradiation, whereby the |*g_lum_* | value will be tuned with the template helicity and the PLQY value be determined by the shell luminophores (e.g., all‐inorganic metal halide perovskites^[^ ^82^ ^]^) and the NH/luminophore interfaces. On the other hand, CPL scattered from the NH@luminophores can then interact with the plasmonic NPs helically assembled at the NH@luminophore surfaces to resonantly enhance the localized *C* of superchiral near fields, especially at the inter‐NP chiral hot spots. Composition of the shell luminophores will be engineered to make the scattered CPL on resonant with the superchiral near fields in the vicinity of helically assembled metal NPs. Molecular substrates/intermediates adsorbed on the plasmonic NPs will experience enhanced symmetry breaking, particularly at inter‐NP chiral hot spots. This strategy holds promise for advancing CPL‐induced photosynthesis of enantiopure enantiomers through symmetry breaking mediated by chiral nano‐inorganics —an area that remains largely underdeveloped.

### 6.3 Electron Spin

Owing to the CISS effect, electrons with spin polarization will be enantioselectively injected into or withdrawn from chiral reactants/ intermediates, and the enantioselectivity is essentially determined by the molecular handedness. It leads to the spin‐selective asymmetric reactions, whereby electron spin enables symmetry breaking. Enhancing the CISS effect is highly desired to amplify the enantiopreference in spin‐selective chemical synthesis. The CISS effect is possible to be enhanced through imposing the helicity onto magnetic nanostructures (e.g., the fabrication of magnetic NHs through glancing angle deposition), and coupling the chiroplasmonic‐induced amplification of *C* with the CISS effect. However, to fully understand the CISS principles, the contributions from spin‐spin and electron‐photon interactions should be investigated. Further demonstrations of enantioselective synthesis via CISS‐induced symmetry breaking will accelerate the advancement of spin chemistry.

It should be emphasized that electron spin is not an isolated phenomenon but may serve as a unifying mechanism underlying various chiral effects. For example, the CISS effect reveals that **spin**‐polarized electrons interact enantioselectively with chiral structures, and this principle may be extended to explain the enantioselective adsorption of molecules on chiral surfaces. Additionally, it is indicated that **CPL** could enhance the CISS effects by inducing coherent spin alignment, potentially offering a dual control pathway through both electronic and photonic chirality. Such synergies remain underexplored and could potentially lead to a new type of spin‐controlled photochemical processes.

### 6.4 Origin of Homochirality

Homochirality underpins asymmetric biochemical processes, reactions, and functions, playing a pivotal role in biological systems. However, the processes whereby biological homochirality arose on the prebiotic Earth from achiral or racemic precursors remain ambiguous. It is generally accepted that homochirality does not have a biogenic origin^[^ ^83^ ^]^ but may instead stem from a random or determinate mechanism. The former randomly generates either enantiomer of a chiral compound, whereas the latter forms a single enantiomer with predetermined handedness. Symmetry breaking followed by chirality amplification is now widely regarded as a plausible explanation for the emergence of biological homochirality.^[^ ^84^ ^]^

Before the emergence of organic molecules, the prebiotic Earth was composed primarily of inorganic minerals, and life is believed to have originated in the ocean. Some minerals were chiral crystals, and biological homochirality has been proposed to stem from asymmetric chemical processes occurring at the chiral surfaces of mineral crystals, particularly in marine environments.^[^ ^85^ ^]^ As chiral minerals were gradually eroded by seawater, they produced chiral nanominerals, which may have acted as effective stimuli to trigger symmetry breaking. However, efforts to investigate symmetry breaking stimulated by chiral nano‐minerals have slowly progressed, mainly owing to the overwhelming fabrication of chiral nano‐inorganics through chirality transfer from organic chiral ligands.^[^ ^86^ ^]^ Recent studies have provided compelling evidence that the atomic‐scale chiral arrangements at mineral surfaces can directly induce the enantioselectivity. For example, Li et al. reported that natural pyrite surfaces could facilitate the prebiotic enantiopreferential formation of D‐amino acid, suggesting that chiral features intrinsic to mineral surfaces may have guided the early selection of biomolecular handedness.^[^ ^87^ ^]^ This finding inspires the hypothesis that chiral nano‐inorganics—especially those with chiral surfaces, edges, and mesostructures—may have similarly contributed to molecular symmetry breaking and the prebiotic enantioselection. Organic chiral ligands, covering chiral nano‐inorganics, have been found to effectively manipulate symmetry breaking of biomolecular building blocks through intermolecular enantiospecific interactions; consequently, the intrinsic contribution of the inorganic component is often obscured. The success in generating organic‐free chiral nano‐inorganics (Figure 1) paves the way to explore the origin of homochirality exclusively contributed from chiral nano‐inorganics through the triggering of symmetry breaking.

Chiral nano‐minerals are predicted to have been dispersed throughout prebiotic seawater, where symmetry breaking could be stimulated while being exposed to high temperature (due to an eruption of volcano), strong solar illumination and electrochemical stimulation (owing to lightning). Experimental evidence further supports the natural occurrence of chiral nano‐inorganics. For instance, Chan et al. demonstrated that magnetite plaquettes found in meteorites exhibit inherent asymmetry, implying that chiral nano‐inorganics can indeed be formed under natural geochemical conditions.^[^ ^88^ ^]^ Under these harsh prebiotic environments, organic‐free chiral nano‐inorganics could provide an appropriate venue where molecular symmetry could be effectively broken ascribed to the enantiospecific inorganic–organic interactions, chirality transfer from CPL to molecules, and spin‐selective chemistry. Notably, recent theoretical modeling supports the idea that an avalanche‐like symmetry‐breaking mechanism driven by the CISS effect could have occurred under the prebiotic conditions, providing a new perspective on how the spin‐dependent processes might have amplified the chiral stimuli in the prebiotic chemistry.^[^ ^89^ ^]^ This will shed light on the origin of biological homochirality—an unresolved mystery that fundamentally hinders our understanding of asymmetric biochemical processes and functions, which are essential to public health and sustainable environmental development.

## Conflict of Interest

The authors declare no conflict of interest.
